# Maladie de Castleman avec manifestation systémique: à propos d’un cas

**DOI:** 10.11604/pamj.2025.52.84.48274

**Published:** 2025-10-24

**Authors:** Ahlam Hmimsa, Nabil Touihem, Hicham Attifi, Mounir Hmidi

**Affiliations:** 1 Service d'Oto-Rhino-Laryngologie et de Chirurgie Cervico-Faciale, Hôpital Militaire Moulay Ismail, Meknès, Maroc

**Keywords:** Maladie de Castleman, polyadénopathie, interleukine-6, syndrome de Raynaud, cas Clinique, Castleman disease, polyadenopathy, interleukin-6, Raynaud's phenomenon, case report

## Abstract

La maladie de Castleman est une pathologie lymphoproliférative rare. Elle se manifeste par une lymphadénopathie et peut être associée à des manifestations systémiques parfois sévères. Elle peut se présenter sous forme unicentrique, limitée à un seul ganglion ou à une seule région ganglionnaire, et est généralement de bon pronostic. Son traitement repose essentiellement sur l'exérèse chirurgicale, ou multicentrique plus sévère, caractérisée par une atteinte ganglionnaire diffuse et une hyperproduction de cytokines, notamment l'Interleukine-6 (IL-6), expliquant les manifestations systémiques. Le traitement fait appel aux anticorps monoclonaux ciblant l'IL-6, aux immunosuppresseurs, à la chimiothérapie ou à la corticothérapie en fonction de l'étiologie. Elle peut être associée à l'infection par le virus de l'herpès humain type 8 (HHV-8), notamment chez les patients immunodéprimés. Nous rapportons le cas d'un patient de 65 ans présentant une polyadénopathie associée à un syndrome de Raynaud. L'imagerie a mis en évidence des adénopathies multiples ainsi qu'une hépatosplénomégalie. Une cervicotomie exploratrice a été réalisée, suivie d'une analyse anatomopathologique et d'un examen immunohistochimique, ayant permis de poser le diagnostic de maladie de Castleman multicentrique. Ce cas clinique illustre l'importance de considérer cette pathologie dans le cadre du bilan étiologique des polyadénopathies inexpliquées, en particulier lorsqu'elles s'accompagnent de manifestations systémiques atypiques.

## Abstract (en)

Castleman's disease is a rare lymphoproliferative disorder. It typically presents with lymphadenopathy and may be associated with sometimes severe systemic manifestations. It can occur in two clinical forms: the unicentric form, which is limited to a single lymph node or lymph node region, generally has a favorable prognosis and is primarily treated with surgical excision; and the multicentric form, which is more severe, characterized by diffuse lymph node involvement and excessive cytokine production, particularly interleukin-6 (IL-6), which accounts for the systemic symptoms. Treatment in the multicentric form includes monoclonal antibodies targeting IL-6, immunosuppressants, chemotherapy, or corticosteroids, depending on the underlying cause. The disease may be associated with human herpes virus 8 (HHV-8) infection, especially in immunocompromised patients. We report the case of a 65-year-old patient presenting with polyadenopathy and Raynaud's phenomenon. Imaging revealed multiple lymphadenopathies along with hepatosplenomegaly. An exploratory cervicotomy was performed, followed by histopathological examination and immunohistochemical analysis, which confirmed the diagnosis of multicentric Castleman disease. This clinical case underscores the importance of considering Castleman disease in the differential diagnosis of unexplained polyadenopathies, particularly when accompanied by atypical systemic manifestations.

## Introduction

La maladie de Castleman, aussi appelée hyperplasie angiofolliculaire, est une entité rare d'hyperplasie lymphoïde. Elle peut se présenter sous deux formes cliniques principales: la forme unicentrique localisée et la forme multicentrique, systémique. Cette dernière est souvent associée à des symptômes généralisés et à une production excessive de cytokines, notamment IL-6 [1]. Nous présentons ici le cas d'un patient de 65 ans avec une forme multicentrique symptomatique.

## Patient et observation

**Informations du patient:** monsieur MC, âgé de 65 ans, ayant comme antécédents une insuffisance cardiaque chronique traitée par Aldactone® et Lasilix®. Il a consulté initialement au service des urgences pour une douleur abdominale diffuse rebelle au traitement évoluant depuis plusieurs mois. Un scanner abdominal, réalisé dans ce contexte, a permis d'éliminer une urgence chirurgicale et a révélé la présence d'adénopathies mésentériques multiples ainsi qu'une hépato-splénomégalie homogène. L'évolution est marquée par l'apparition d'une polyadénopathie généralisée, associée à des épisodes de syndrome de Raynaud caractérisés par des changements de couleur des doigts et des orteils au froid. Aucune fièvre ni de sueurs nocturnes n'ont été rapportées.

**Figure 1:**
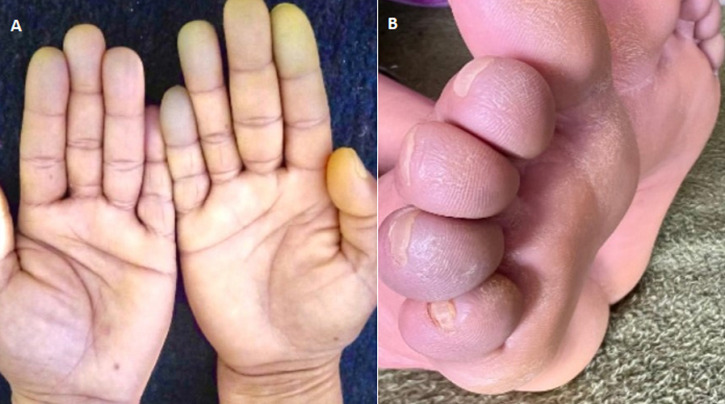
A,B) phénomène de Raynaud

**Résultats cliniques:** à l'examen clinique, on notait des adénopathies palpables, fermes, mobiles, indolores, principalement cervicales, axillaires et inguinales, mesurant entre 1 et 2 cm, sans signe inflammatoire local. L'examen trouve également des doigts et des orteils gonflés, blancs, cyanosés, froids et insensibles (Figure 1).

**Démarche diagnostique:** devant le tableau clinique et le contexte épidémiologique, plusieurs diagnostics ont été envisagés initialement: lymphome non hodgkinien, tuberculose, sarcoïdose, infections virales: Epstein-Barr virus (EBV), cytomegalovirus (CMV), *Human Immunodeficiency Virus* (VIH) et la maladie auto-immune (lupus, syndrome lymphoprolifératif).

**Figure 2:**
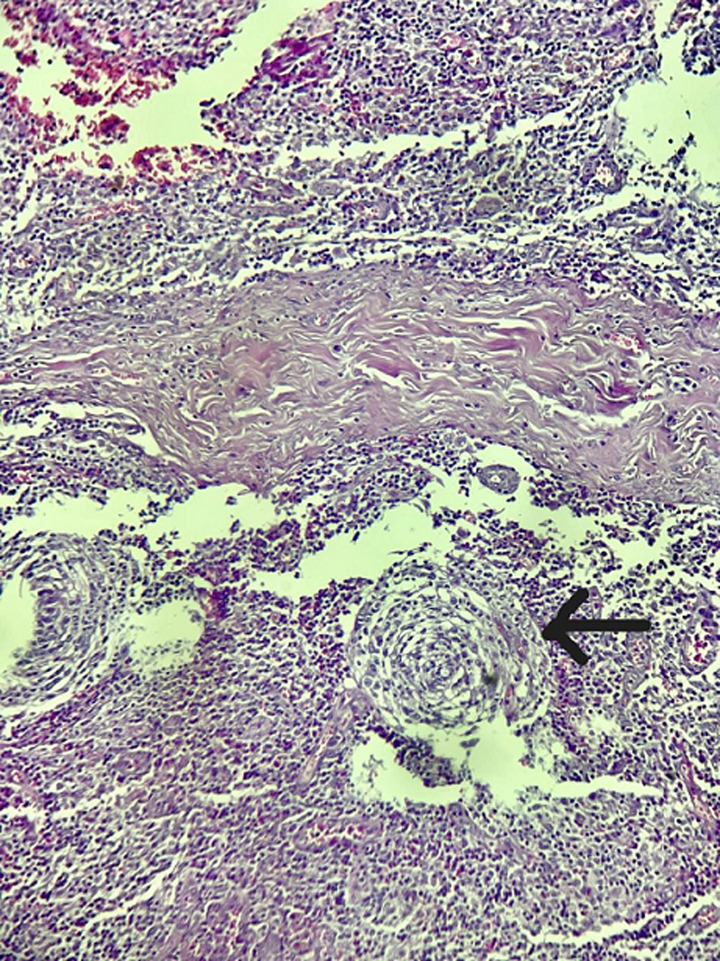
parenchyme ganglionnaire avec une zone de manteau épaissie et des lymphocytes disposés en couches donnant l'aspect en bulbe d'oignon (G x 20)

**Figure 3:**
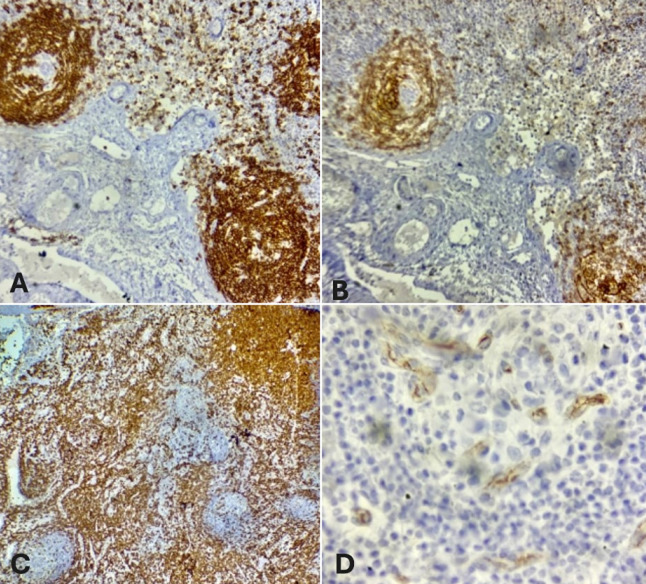
images microscopiques après marquage immunohistochimique: A) marquage intense des lymphocytes B folliculaires pour le CD20; B) marquage intense des cellules dendritiques folliculaires péri vasculaires pour le CD23; C) marquage intense du fond tumoral riche en lymphocytes T par le CD3; D) marquage de cellules tumorales TDT positives autour des zones en bulbe d'oignon

**Figure 4:**
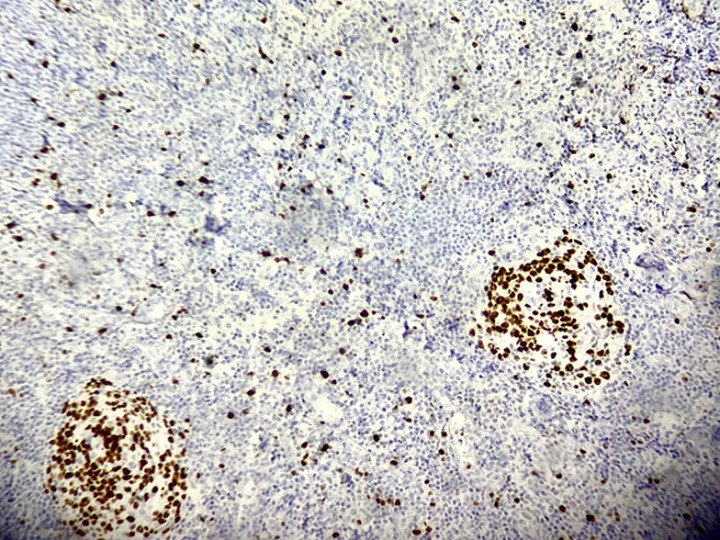
image microscopique après marquage immunohistochimique par Ki-67 montrant un index de prolifération élevé au sein des centres germinatifs

Le bilan biologique complet incluant ainsi les sérologies (notamment celles de HHV-8, VIH, CMV et EBV) et l'exploration immunologique s'est révélé sans anomalie significative en dehors d'une élévation de la C-reactive protein (CRP) et d’une anémie inflammatoire. Le scanner cervico-thoraco-abdomino-pelvien a montré des adénopathies multiples: cervicales, axillaires, médiastinales, abdominales et inguinales, associées à une hépatosplénomégalie homogène. Une cervicotomie exploratrice a été réalisée. L'analyse histologique de l'adénopathie réséquée a mis en évidence une hyperplasie folliculaire et vasculaire avec un aspect caractéristique en bulbe d'oignon (Figure 2). L'étude immunohistochimique a montré une positivité pour le cluster de différenciation 3, 20, 23 (CD3, CD20, CD23) et le terminal désoxynucléotidyl transférase (TDT) (Figure 3), avec un index de prolifération Ki-67 élevé (Figure 4). L'ensemble des données cliniques, radiologiques et histologiques a permis de poser le diagnostic de maladie de Castleman multicentrique idiopathique à HHV-8 négatif.

**Intervention thérapeutique:** le patient a été orienté vers le service de médecine interne pour une prise en charge thérapeutique spécialisée, où un traitement par tocilizumab 8 mg/kg toutes les 2 semaines a été initié, conformément aux recommandations actuelles pour la forme idiopathique multicentrique de la maladie de Castleman. La tolérance et la réponse clinique ont été surveillées régulièrement par un suivi biologique et radiologique.

**Suivi et résultats:** *suivi en cours:* bonne observance et tolérance du traitement, amélioration partielle des symptômes rapportés, pas de complications immédiates notées. La CRP montre une cinétique de baisse progressive.

**Consentement éclairé du patient:** le patient a donné son consentement oralement pour la publication de ce cas.

## Discussion

La maladie de Castleman est une pathologie rare dont l'origine exacte n'est pas entièrement élucidée [1], décrite initialement par Castleman *et al*. dans les années 1950 [2]. On distingue deux formes cliniques principales: unicentrique, limitée à un seul ganglion ou à une seule région ganglionnaire, et multicentrique, plus rare, impliquant plusieurs ganglions et pouvant s'accompagner de manifestations systémiques. Elle peut être associée à l'infection par le virus HHV-8 [3], notamment chez les patients immunodéprimés, ou être de forme idiopathique [4]. Les manifestations systémiques au cours de cette maladie peuvent être expliquées par la production excessive de cytokines, notamment l'interleukine-6. Ce qui peut contribuer à divers phénomènes paranéoplasiques et à une dérégulation du système immunitaire avec une dysfonction endothéliale menant à des spasmes vasculaires qui se manifestent par le syndrome de Raynaud [5,6].

Le diagnostic de la maladie de Castleman repose principalement sur l'analyse histologique des ganglions lymphatiques. Les éléments caractéristiques incluent une hyperplasie folliculaire, une vascularisation proliférante ainsi qu'un aspect en “bulbe d'oignon” dans les centres germinatifs, notamment dans les formes hyalino-vasculaires [2,5]. L'immunohistochimie est indispensable pour confirmer le diagnostic, montrant typiquement une positivité des marqueurs lymphocytaires: CD3 (cellules T), CD20 (cellules B), CD23 (centres germinatifs), un index Ki-67 élevé, traduisant une forte prolifération cellulaire, et une positivité du CD34 témoignant d'une néoangiogenèse importante. Il est crucial d'identifier la forme exacte de la maladie, car la prise en charge thérapeutique et le pronostic diffèrent radicalement [7].

Le traitement de la forme multicentrique dépend de l'étiologie et de la sévérité clinique, et il est basé sur le rituximab en première ligne pour les formes associées au HHV-8 [4,6]. Seuls ou en association avec une chimiothérapie, les antagonistes de l'IL-6 sont le traitement de choix pour les formes actives idiopathiques comme le siltuximab et le tocilizumab [6,7], et dans les formes sévères ou réfractaires, une corticothérapie, des immunosuppresseurs ou une chimiothérapie peuvent être utilisés, avec un suivi long rigoureux pour surveiller les récidives. Par contre, la forme unicentrique est généralement bénigne et peut être traitée efficacement par l'exérèse chirurgicale complète du ganglion atteint, avec un excellent pronostic [3,8].

## Conclusion

La maladie de Castleman, bien que rare, doit être envisagée dans le diagnostic différentiel des adénopathies inexpliquées, notamment lorsqu'elles sont volumineuses, persistantes et atypiques. Le diagnostic repose sur l'histologie. Le traitement repose principalement sur la chirurgie dans les formes unifocales, avec un excellent pronostic en l'absence de signes systémiques. Toutefois, les formes multicentriques nécessitent une prise en charge multidisciplinaire et individualisée. Ce cas souligne l'importance de la collaboration entre cliniciens et anatomopathologistes afin d'optimiser la prise en charge de cette pathologie rare.
